# The central role of perceived control for reducing anxiety among mothers of NICU hospitalized preterm babies

**DOI:** 10.1192/j.eurpsy.2023.1067

**Published:** 2023-07-19

**Authors:** M. Kestler-Peleg

**Affiliations:** School of Social Work, Ariel University, Israel

## Abstract

**Introduction:**

Mothers of preterm babies that were hospitalized in Neonatal Intensive Care Unit (NICU), might react with anxiety due to the extremely challenging circumstances. From maternal point of view, after a sudden, high-risk and traumatic birth that has recently occurred, their fragile preterm babies are in an intimidating medical environment, separated from them and are at high-risk of morbidity and mortality. Those circumstances, accompanied by great uncertainty, provoke perceptions of control such as locus of control and maternal self-efficacy, which are both known as central in evoking anxiety.

**Objectives:**

The proposed presentation examines the mediating effect of maternal self-efficacy in the association between locus of control and anxiety, among mothers of hospitalized babies in the NICU, above and beyond gestational age and mothers’ socio-economic status.

**Methods:**

The participants in the present study were 128 Israeli mothers of 208 NICU hospitalized preterm babies. They completed self-report questionnaires regarding their background variables, internal locus of control, maternal self-efficacy, and anxiety.

**Results:**

The analysis showed that while gestational age and mothers’ socio-economic status were controlled, internal locus of control had a significant and positive effect on maternal self-efficacy, and that maternal self-efficacy had a significant and negative effect on mothers’ anxiety. Finally, the direct negative effect of internal locus of control on anxiety decreased with the inclusion of maternal self-efficacy. Altogether, the model explains 26.2% of the variance in maternal anxiety.

**Conclusions:**

The results demonstrate the central role of senses of control in reducing the levels of anxiety experienced by mothers while their preterm babies are NICU hospitalized. fessionals’ interventions with preterm mothers should focus on supporting and encouraging perceptions of internal control as well as promote positive self-perception regarding their ability to succeed in mothers’ tasks. Higher internal perception of control will lead to higher maternal confidence in their ability to perform as mother, which in turn will reduce their anxiety levels. These outcomes may potentially benefit not only the preterm mothers themselves, but also their babies through higher levels of maternal engagement and responsibility as well as through better maternal functioning and subjective well-being. These will impact infants in the short and long run, in terms of physical, cognitive and emotional development.

**Disclosure of Interest:**

None Declared

